# Application of Artificial Intelligence Technologies as an Intervention for Promoting Healthy Eating and Nutrition in Older Adults: A Systematic Literature Review

**DOI:** 10.3390/nu17203223

**Published:** 2025-10-14

**Authors:** Kingsley (Arua) Kalu, Grace Ataguba, Oyepeju Onifade, Fidelia Orji, Nabil Giweli, Rita Orji

**Affiliations:** 1 Food Science & Nutrition, School of Science, Translational Health Research Institute, Western Sydney University, Richmond, NSW 2753, Australia; 2 Faculty of Computer Science, Dalhousie University, Halifax, NS B3H 4R2, Canada; grace.ataguba@dal.ca (G.A.); fidelia.orji@dal.ca (F.O.); rita.orji@dal.ca (R.O.); 3 School of Medicine and Public Health, University of Newcastle, Callaghan, NSW 2308, Australia; peju.onifade@newcastle.edu.au; 4 School of Computer, Data and Mathematical Sciences, Western Sydney University, Sydney, NSW 2751, Australia; n.giweli@westernsydney.edu.au

**Keywords:** artificial intelligence, nutrition, health, older adults

## Abstract

**Background/Objectives:** The aging population faces a multitude of health challenges, particularly when it comes to maintaining proper nutrition. Age-related physiological changes, such as decreased metabolism, diminished taste perception, and difficulty in chewing, can lead to insufficient nutrient intake, ultimately resulting in malnutrition. It is crucial to address these issues to promote not only physical health but also overall well-being. In this modern era, artificial intelligence (AI) technologies, including robots and machine learning algorithms, are being increasingly harnessed to encourage healthy eating habits among older adults. This is critical to support healthy aging and mitigate diet-related chronic diseases. However, little or no synthesis has established their effectiveness in delivering personalized, scalable, and adaptive interventions for older adults. This systematic review considers the state-of-the-art application of AI-based interventions aimed at improving dietary behaviors and nutritional outcomes in older adults. **Methods:** Following the PRISMA 2020 guidelines and a registered PROSPERO protocol (ID: CRD420241045268), we systematically analyzed 30 studies we collected from five databases, published between 2015 and 2025 based on different AI techniques, including machine learning, natural language processing, and recommender systems. We synthesized data collected from these studies to examine the intervention types, outcomes, and methodological approaches. **Results:** Findings from our review highlight the potential of AI-based interventions to promote engagement among older adults and improve adherence to healthy eating guidelines. Additionally, we found some challenges related to ethical concerns such as privacy and transparency, and limited evidence of their long-term effectiveness. **Conclusions:** AI-based interventions offer significant promise in promoting healthy eating among older adults through personalized, adaptive, and scalable interventions. Yet, current evidence is constrained by some methodological limitations and ethical concerns, which calls for future research to design inclusive, evidence-based AI interventions that address the unique physiological, psychological, and social needs of older adults.

## 1. Introduction

The aging population faces the risk of age-related diseases, including malnutrition and choking, which shows the importance of promoting healthy eating to improve their quality of life [1,2]. This is further justified by research showing a significant increase in the global burden of non-communicable diseases (NCDs), such as heart disease, diabetes, and chronic respiratory conditions, particularly among older adults, resulting in higher healthcare expenditures for both individuals and health systems worldwide [3]. Most recent reports from the World Health Organization (WHO) show that, by 2050, 2.1 billion older adults [4] will be affected. AI technologies within the context of this review focus on systems that apply machine learning, natural language processing and recommender systems, including AI-integrated assistive technologies aimed at promoting healthy eating behaviors among older adults. AI technologies present innovative solutions aimed at fostering healthier eating habits among older adults. However, several significant gaps must be addressed to maximize their effectiveness. A common shortcoming among many existing tools is their lack of personalization [5]. For instance, these platforms often fail to tailor dietary recommendations based on individual health conditions, such as diabetes, hypertension, or heart disease, which are frequently diagnosed in this age demographic [6]. This oversight can lead to the provision of generic advice that may not align with the specific medical needs or dietary restrictions of older adults.

Moreover, cultural sensitivity is often overlooked, resulting in suggested meal plans that may not resonate with the diverse backgrounds and culinary preferences of users [7]. This can render the recommendations ineffective or unappealing, diminishing the user’s motivation to adopt healthier eating habits [7]. Accessibility is another pressing issue; many older adults face challenges due to limited digital literacy, making it difficult for them to operate complex applications that could otherwise provide valuable support in their dietary choices [8]. AI is transforming global nutrition and public health through ethical, evidence-based innovation. The FAO, IBM, and Microsoft co-signed the Rome Call for AI Ethics to promote human-centric digital progress in agri-food systems [9]. Nutrition International and Atlas AI use geospatial machine learning to identify underserved households and improve climate-resilient nutrition programs in low- and middle-income countries [10]. Meanwhile, the IUNS Task Force on Precision Nutrition advances AI-enabled approaches like nutrigenomics and metabolomics to personalize dietary recommendations and improve health outcomes [11]. These initiatives highlight the global commitment to responsible AI in nutrition science.

The current landscape also reveals a limited integration of AI technologies within clinical care settings [12,13,14]. Healthcare professionals often depend on traditional methods of dietary management, which can fall short of adequately addressing the unique nutritional needs of older patients [15]. Additionally, there is a significant concern regarding the long-term efficacy of these AI technologies in enhancing dietary habits and improving overall health outcomes [16]. Evidence supporting the sustained impact of these tools is scarce, raising doubts about their reliability.

Data privacy emerges as another critical concern, particularly given the sensitive nature of health-related information [17]. The underrepresentation of older adults in the datasets used to train these AI systems exacerbates the problem, potentially leading to less reliable and relevant outcomes for this age group [18].

In light of these challenges, the research community is making concerted efforts to develop AI technologies that are specifically designed to improve healthy eating behaviors and reduce the risk of nutrition-related diseases among older adults [19]. This includes the creation of more inclusive AI models that consider a broader spectrum of dietary needs and preferences. Additionally, there is a focus on enhancing user interfaces to ensure that these tools are more accessible to individuals with varying levels of digital literacy [20]. Equally important are the ongoing efforts to implement robust privacy measures to protect sensitive health data [21].

Researchers aim to address the barriers currently inhibiting the effectiveness of AI in promoting healthier eating habits, ultimately working towards better health outcomes for the older population in a more competent and compassionate manner [22]. These technological innovations have shown potential opportunities for delivering continuous, personalized care to older adults [23,24,25]. Targeting the elderly demographic is particularly important given these rising trends of malnutrition and related complications, such as choking [26]. Recent achievements in this area have been realized through the utilization of pretrained language models, such as Bidirectional Encoder Representations from Transformers (BERT) and Generative Pre-trained Transformer 2 (GPT-2), developed by Google and OpenAI, respectively [27]. The application of AI technologies marks a significant transition from traditional methods of nutritional assessment and care, enabling more precise evaluations of body weight, food intake, dietary habits, and eating behaviors among older adults [28]. Novel AI interventions like wearable devices tracking health markers and smartphone programs providing personalized dietary advice have also been proposed to enhance nutritional status among the elderly [29]. Despite the ethical concerns related to privacy and data security, AI nonetheless holds great promise to encourage healthy eating behaviors and nutritional care among the elderly [30]. Specifically, applications of machine learning and deep learning algorithms can increase the accuracy and efficiency of diet estimation and monitoring of eating behaviors significantly [31,32]. However, despite the numerous publications on AI technologies and their implementation in enhancing nutrition and health outcomes, there are continuous controversies regarding their validity and performance [6,33]. This review aims to examine how artificial intelligence (AI), a combination of methods, techniques, and technologies designed to analyze eating behaviors, generate tailored dietary recommendations, and deliver personalized nutrition support, has been used as an intervention to promote healthy eating and nutrition among older adults. The review adopts an exploratory approach, recognizing that the field of artificial intelligence is vast and continually evolving. Accordingly, the scope of this review reflects the AI interventions and technologies that have been empirically examined within existing studies on healthy eating and nutrition among older adults up to the time of review. Although heterogeneous age group studies were considered, we reported our findings for individuals aged 60 and above, which is the World Health Organization’s working definition of older adults [34]. To guide this research, we propose five (5) research questions (RQs), as follows:

- What are the different trends in these studies? (RQ1).
- How have the different AI technologies been used to promote healthy eating habits among older adults? (RQ2).
- How effective are AI technologies for promoting healthy eating habits among older adults? (RQ3).
- What are the current limitations and ethical implications of designing AI technologies for promoting healthy eating habits among older adults? (RQ4).
- What are the future implications of designing AI technologies as nutrition-based interventions for older adults in future research? (RQ5).

Our study contributes to knowledge in three ways. First, we provide a systematic synthesis of trends in how AI has been applied to nutrition interventions for older adults. Second, we highlight effective patterns, limitations, and ethical concerns surrounding the design and deployment of these systems. Finally, we outline future design and research directions, offering a roadmap for inclusive, ethical, and impactful AI-driven nutrition technologies targeting older populations.

## 2. Background and Related Work

Although numerous reviews have been broadly considering the ability of various technologies to support healthy eating, only a few have explicitly targeted AI-assisted systems specifically intended to be used by older adults. For example, Aggarwal et al. [35] assessed the feasibility and efficacy of AI-assisted chatbots in modifying health behaviors. Their findings suggested chatbots can enable healthy habits, smoking cessation, medication adherence, and reduced drug use through goal setting, real-time feedback, and behavior monitoring. Oh et al. [36] conducted a comprehensive review that examined the various characteristics and functionalities of artificial intelligence chatbots, specifically their effectiveness in promoting changes in physical activity, dietary habits, and weight management behaviors among users. In a different vein, Sosa et al. [37] provided a thorough review addressing the overarching delays and shortcomings in the application of AI technology within the field of nutrition. Meanwhile, Starke and co-researchers research focused on the potential of AI to deliver personalized dietary advice aimed at encouraging healthier cooking practices at home [38]. Notably, however, none of these studies or reviews specifically targeted the unique dietary challenges faced by older adults, a demographic that may benefit significantly from tailored nutritional guidance.

Chew et al. [39] gave a scoping review, highlighting the promise of AI for managing eating behavior and enhancing adult weight loss. The review highlighted three mechanisms: self-monitoring, goal setting, and self-control. Despite conceptual richness, there were limited interventions that implemented real-time personalized micro-interventions through AI. Besides, older participants were not well represented in the studies included, and from this review, it was ascertained that, while AI holds promise, its application to sustainable weight loss is undeveloped. Zheng et al. [40] also carried out another scoping review whose goal was to map AI tools for assessing dietary intake. The authors determined that AI models, especially those that utilized image recognition and wearable sensors, improved the accuracy of food detection and estimation of nutrients. Although there were some interventions that used advanced modalities such as jaw motion sensors and RGB-D imaging, the review pointed out shortcomings in using these technologies for a variety of populations, including older adults with sensory or cognitive impairments.

There were also broader discussions provided on AI in nutrition by Sosa-Holwerda et al. [37], who elaborated on the uses of AI across various fields of nutrition-related fields. Diet assessment was the dominant application, though fewer studies have examined AI for behavioral interventions or its value to support malnutrition among older adults. The review also highlighted key issues regarding data privacy, algorithmic fairness, and the ethical aspects of AI application to vulnerable populations. In addition, Robert et al. [41] meta-analyzed eHealth interventions in middle-aged and older adults. Although most interventions demonstrated clinical improvements, including weight and cholesterol decreases, behavior changes such as the consumption of more fruit and vegetables were less frequently achieved. Most of the interventions did not incorporate age-sensitive personalization, a glaring error given that digital literacy barriers are frequently confronted by older adults.

Finally, Singh et al. [42] meta-analyzed chatbot interventions to affect lifestyle habits. Statistically significant increases in physical exercise, fruit and vegetable consumption, and sleeping patterns were supported by their findings. Text and AI-based chatbots were found to outperform voice-only systems, while multicomponent interventions outperformed chatbot-alone interventions. Yet, the study cited the absence of subgroup analysis among older adults and consequently its inability to make the findings applicable to this subgroup. While there is plenty of evidence in the literature to demonstrate the potential and feasibility of AI-based food technologies, few reviews have evaluated their application systematically across aging groups or considered the full range of behavioral, technical, and ethical issues needed for effective implementation. This review seeks to fill this gap through a synthesis of the state-of-the-art application of AI-based nutritional technologies specifically created for older adults, discussing behavior monitoring approaches, design considerations, and limitations to inform future development and implementation.

## 3. Methodology

We present the methodology we employed in our literature review search. This covers the selection criteria, search procedures, and analysis. We ensured this review focused only on older adults by applying inclusion criteria that restricted eligible studies to those involving participants aged 60 years and above, in line with the World Health Organization’s definition of older adulthood [43]. Although some studies included mixed age groups, we extracted and analyzed only the outcomes relevant to participants within this age range.

### 3.1. Selection Criteria

Our selection of articles was guided by pre-determined inclusion and exclusion criteria of relevance, quality, and correspondence to the research objective. Studies were considered eligible for inclusion if they met the following: (1) population: human participant studies of the target population (older adults with or without specified health conditions); (2) intervention/technology: studies involving artificial intelligence related technologies, e.g., chatbots and sensors to monitor or aid in nutrition; (3) study design: empirical studies employing qualitative, quantitative, or mixed methods, i.e., experimental, quasi-experimental, or observational design; (4) publication type: peer-reviewed journal articles or conference proceedings published in the English language; and (5) time frame: studies published between 2015–2025 to reflect up-to-date and state-of-the-art technological applications. On the other hand, articles were excluded if they: (1) were theoretically grounded but lacked empirical analysis; (2) lacked study result details or implementation strategies; (3) were review papers, case studies, book chapters, or position papers. These filtering criteria ensured that only empirically supported and relevant articles progressed to the final analysis phase, as per the aims of this review. The search was conducted in April 2025. To ensure rigor and minimize bias, one author conducted the initial screening of all retrieved papers by reviewing titles, abstracts, and full texts against the predefined inclusion and exclusion criteria. To ensure reliability, a second researcher independently verified this process by cross-checking the eligibility decisions and confirming the accuracy of the extracted data. Interrater reliability between the two reviewers was assessed using Cohen’s κ (κ = 0.77), indicating substantial agreement [44]. Any disagreements were discussed and resolved through consensus, ensuring that only studies meeting the agreed standards were retained for analysis. This dual-review process, supported by a high level of agreement, strengthened the methodological rigor of the study and minimized the risk of bias in study selection and data extraction.

**Figure 1:**
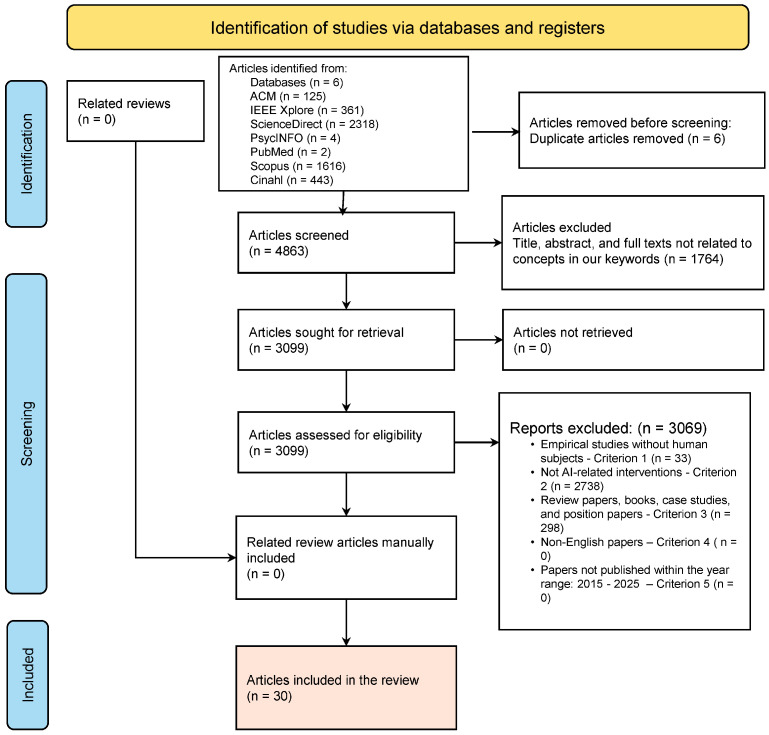
PRISMA diagram showing our paper filtering procedure (French et al. [47]). Appendix A presents a summary of the literature included in this review (N  =  30 studies).

We present details of our inclusion and exclusion criteria in Figure 1, showing the Population, Intervention, Comparison, Outcomes, and Study (PICOS) design guidelines. Specifically, an assessment of artificial intelligence interventions aimed at promoting healthy eating behaviors with or without specific health conditions, including obesity and overweight, was performed. The population focus was generalized to older adults, interventions focused on artificial intelligence (AI) technologies, comparisons and outcomes were guided by prior literature. Overall, empirical findings covering the impact of AI interventions from prior literature were systematically synthesized, revealing predominantly positive effects on dietary behaviors, user satisfaction, and health-related outcomes among older adults, as well as highlighting persistent challenges in real-world implementation and ethical design.

### 3.2. Search Procedures and Data Extraction

For this systematic review, we followed the Preferred Reporting Items for Systematic Reviews and Meta-Analyses (PRISMA) guidelines [45,46], with a comprehensive search using a combination search strings, and Boolean operators: “AI [All Fields]; OR Artificial Intelligence [All Fields]; AND technologies [All Fields]; OR interventions [All Fields]; AND healthy [All Fields]; AND eating [All Fields]; OR nutrition [All Fields]; OR diet [All Fields]; AND older adults [All Fields]; OR elderly [All Fields]; OR aging [All Fields];” The search strategy was applied on seven databases: ACM digital library; IEEE Xplore; ScienceDirect; PsycINFO; CINAHL; PubMed; and Scopus (Figure 1).

The search was expanded to include titles, abstracts, and the full text of papers published between 2015 and 2025. The search process returned a total of 4869 papers distributed across the six (6) databases as follows: ACM digital library (125), IEEE (361), ScienceDirect (2318), PsycINFO (4), CINAHL (443), PubMed (2), and Scopus (1616). We exported the search results from each of the databases in BibTeX format and uploaded them to Rayyan. Rayyan is a free web and mobile tool that facilitates systematic review following a semi-automated screening process (accessed on 21 April 2025, https://www.rayyan.ai/). Rayyan enhances multiple reviews of the screening process, duplicate paper removal, and the application of inclusion/exclusion criteria. After we carefully reviewed the papers, we excluded 6 duplicates. Then, we screened the papers based on the title using all the keywords from our search strings. In addition, we screened the title, removing review-related papers. Following this screening procedure, we excluded 3069 papers. Figure 1 presents the flow diagram of the screening process.

This systematic review was conducted and reported in accordance with the Preferred Reporting Items for Systematic Reviews and Meta-Analyses (PRISMA) 2020 guidelines [45]. The review protocol was registered in the International Prospective Register of Systematic Reviews (PROSPERO; Registration ID: CRD420241045268).

Furthermore, we manually extracted different fields from valid papers. These fields include bibliometric data (such as authors, year of publication, type of publication, database, and geographical location), principles, concepts, theories and models, breakfast, lunch, dinner (and/or snack), justification for food intake, eating patterns, underlying conditions, types of AI technologies, user data collected, target participants, age of participants, gender of Participants, types of sensors, name of application, features of App, evaluation method, study instrument, result, effectiveness, challenges, limitation, duration of the study, no. of participants, and recommendations, machine learning model, accuracy of machine learning models employed, and authors keywords. To ensure that only AI-related interventions were included, the “Types of AI technologies” field extracted from studies ensured that we screened non-AI-related interventions. This approach ensured consistency in classification, transparency in reporting, and alignment with the review’s main objective to focus exclusively on AI-driven interventions. These extracted data from all included studies were entered into a standardized coding sheet. In preparation for data analysis, we cleaned our data to ensure consistency in terminology, resolved minor discrepancies in reported values by confirming data from the specific papers, and standardized data where applicable (e.g., accuracy metrics, sample sizes, health outcomes). Missing or unclear data were recorded as ‘Not Reported/Applicable’ and retained in the dataset to maintain transparency.

### 3.3. Data Analysis

We employed descriptive statistics, i.e., percentages and frequencies, and inferential statistics to assess inter-rater reliability, to analyze data collected from different articles. We employed descriptive statistics specifically while answering research questions RQ1, RQ2, and RQ3. Furthermore, qualitative data collected from these articles were analyzed following a systematic content analysis procedure of Braun and Clarke [48] to explore important themes and patterns across studies we reviewed [49]. We also employed content analysis following Braun and Clarke’s procedure [48] to answer RQ4 and RQ5. This quantitative and qualitative approach together provided an in-depth understanding of the field. We conducted the quantitative analysis using Microsoft Excel and qualitative analysis using NVIVO 14 [50]. In the next section, we report the procedure and inter-rater reliability analysis where applicable.

## 4. Results

In this section, we present key findings from our systematic analysis of the data collected. First, we cover the results from our bibliometric analysis of the papers we included in this review. This bibliometric analysis shows the distribution of papers by year, venue of publication, and geographical region where studies related to the application of AI technologies for older adults were carried out.. Second, we present results on the different trends in these studies (RQ1). Third, we present insights into how different AI technologies have been used for promoting healthy eating habits among older adults (RQ2). Fourth, we present an evaluation of the effectiveness of AI technologies for promoting healthy eating habits among older adults (RQ3). Fifth, we cover the limitations and ethical implications for designing AI technologies for promoting healthy eating habits among older adults (RQ4). Sixth, we present design recommendations for designing AI technologies as nutrition-based interventions for older adults in future research (RQ5).

### 4.1. Bibliometric Analysis and Trends (RQ1)

In this section, we cover bibliometric analysis, showing the trends in concepts, theories and models (Section 4.1.1), and eating patterns tracked using AI technologies (Section 4.1.2).

#### 4.1.1. Distribution of Common Principles, Concepts, Theories and Models for Promoting Healthy Eating and Nutrition in Older Adults

The theoretical and modeling foundations of studies applying AI technologies to promote healthy eating and nutrition among older adults show five broad categories. We conducted a thematic analysis (following Braun and Clarke’s six-step framework) of the theories, concepts, and models reported in the reviewed articles. Both coders independently reviewed the “Principles, Concepts, Theories, and Models” section of each study, generated initial codes (e.g., “feminist theory,” “self-disclosure theory,” “CNN,” “ontology”), and iteratively refined these into broader categories. Disagreements were discussed and resolved through consensus, and interrater reliability was substantial (Cohen’s *κ* = 0.79). The process yielded a large set of raw codes (over 25 individual theoretical or computational approaches), which were then merged into the following five overarching themes:

1. Behavioral and Social Theories: A few studies applied behavioral science principles, theories, and frameworks such as the Self-Disclosure concept, Feminist Theory, or Technology Acceptance Model (TAM) in conceptualizing users’ interaction with AI technologies. These frameworks play a significant role in the comprehension of aging adults’ uses and attitudes towards healthcare-related technologies. For example, Jo et al. [51] used the concept of Self-Disclosure grounded in Social Penetration theory (1973) that holds that one is more likely to share personal or sensitive details in situations that are seen as non-judgmental, friendly, and emotionally supportive [52]. They implemented their intervention via the use of CareCall, a voice-based LLM chatbot used with socially isolated older adults in South Korea. Drawing on the Self-Disclosure theory, they designed the chatbot to remember and make reference to past conversations in terms of long-term memory (LTM), creating continuity and personalization throughout multiple sessions. Their findings showed that repeated LTM-evoked chats caused users to offer more information on different health conditions, such as their eating issues and management strategies. Wu et al. [53] applied Feminist Theory, such as Marjorie DeVault’s “feeding as care work” theory, to theorize the social, gendered, and emotional dimensions of family dinners.

DeVault envisions feeding not just as a series of domestic tasks, but as relational and affective practice embedded in interpersonal care, emotional labor, and cultural expectations. In a co-designing experiment with caregivers and older adults, they made sure the resulting artificial intelligence (AI) systems acknowledge and support the caregiving work involved in eating, valuing users’ resistance, fatigue, and emotional reactions to eating management. Thus, by recognizing feeding as care work, nutrition-interrelated technologies can be cooperative partners in a wide network of emotional and social care, especially among the elderly. Chao and Hass [54] employed the Technology Acceptance Model (TAM) in order to know how older adults adopt dietary-support AI technologies.

TAM, initiated by Davis [55], postulates that user adoption of technology is influenced primarily by two assumptions: perceived ease of use (the degree to which a user feels that using the technology will improve their performance) and perceived usefulness (the degree to which a user believes that the technology will enhance their performance) [37]. In the present study, the aforementioned constructs were subsequently adapted to assess how older individuals adopt mealtime and diet-support AI technology in the home environment. Results indicate that perceived usefulness was strongly correlated with the ability of the system to maintain autonomy in routine nutrition decisions, while ease of use was strongly correlated with interface clarity and voice support. By situating TAM within the context-free framework, Chao and Hass [54] provided thoughtful insight into the ways in which perceived usefulness and ease of use intersect with the values and constraints of older adults, resulting in a more nuanced application of TAM in the creation of AI-based dietary interventions.

2. Machine Learning and Computational Models: Many studies leveraged deep learning architectures such as Convolutional Neural Networks (CNNs), Sequence-to-Sequence models, SimCLR, or Stacked Ensemble Learning. These were generally used to model dietary behavior, predict nutritional needs, or support real-time decision-making through sensor data or user input. For example, Elfert et al. [56] implemented a Convolutional Neural Network (CNN) architecture, specifically SSD-MobileNet V2, to support real-time food recognition as part of a dietary monitoring system for older adults. CNNs are a class of deep learning models particularly suited for processing visual data and are widely used in tasks such as image classification and object detection. They developed a mobile-based application that used the SSD-MobileNet V2 model for detecting and classifying food items in images captured during meals. This development was suitable for older adults, given that it reduced the cognitive burden, leading to a struggle of manually logging foods, and provided a lightweight solution that was suitable for deployment on resource-constrained mobile devices commonly used by older adults.

Patino-Alonso et al. [57] implemented ensemble learning techniques, specifically a stacked ensemble model (AdXGRA) that combined multiple base learners such as AdaBoost, Extreme Gradient Boosting (XGB), Random Forest (RF), Multilayer Perceptron (MLP), and Generalized Linear Models (GLM), to predict and monitor dietary habits and adherence to healthy nutrition among older adults. Ensemble learning is a machine learning paradigm that integrates predictions from multiple models to improve generalization and robustness. By employing ensemble learning, the system enhanced reliability in dietary assessments, key for older adults who may have complex or variable eating patterns.

He et al. [58] utilized a Support Vector Machine (SVM) with Gaussian Kernel to classify eating behaviors based on acoustic and spectral features extracted from meal-related audio data. SVMs are supervised learning models used for classification tasks that aim to find an optimal hyperplane to separate data points into distinct categories. The Gaussian (RBF) kernel enhances the SVM’s ability to handle non-linear relationships by mapping input features into a higher-dimensional space where they become linearly separable. He and colleagues developed an AI-driven system designed to monitor food intake behavior in older adults using non-invasive audio signals captured during eating episodes. The SVM was trained on a feature-rich dataset that included acoustic features such as Pitch/Energy Trajectories (PET), Full Spectrogram Contours (FS-Conts), and Log Symmetric Spectral Difference Levels (LSSDL), along with novel features like Crucial Energy Coefficients (C-coes) and Local Dynamic Features (LDF). By leveraging the Gaussian kernel, the model effectively captured complex variabilities in audio features, essential for estimating portion size and detecting abnormal eating patterns that could signal undernutrition or dysphagia in elderly populations. We elaborate on the accuracy values of these models in Section 4.3.

3. Knowledge-Based and Hybrid Models: Studies we reviewed presented knowledge graphs, ontology-based reasoning, and recommendation systems that integrate rule-based logic and learning modules for personalizing nutrition interventions. For example, in a study conducted by Xu et al. [59], the knowledge graph (KG) methods were used to enhance the semantic interpretation and personalization of dietary interventions for older adults. A knowledge graph is a structured representation of facts and relationships, where entities (e.g., foods, nutrients, diseases) are linked through semantically meaningful connections. This approach enabled the system to identify foods that were consumed, while providing them with explanations of how those choices accorded with broader health contexts, such as managing hypertension, diabetes, or nutritional deficiencies common among older adults. For instance, if the user had a low potassium diet and a history of hypertension, the system could recommend potassium-rich foods while omitting those with excessive sodium. Prasetyo and Baizal [60] utilized ontology-based modeling coupled with Semantic Web Rule Language (SWRL) for creating a nutrition recommendation system for the elderly.

Like the knowledge graphs, artificial intelligence ontologies are formal representations of domain knowledge that define key concepts (e.g., categories of food, nutrients, health outcomes) and relationships between them. Using ontologies, systems can reason about data in a human-understandable, semantically relevant manner, allowing for more intelligent and adaptive health interventions. To represent dietary rules, nutritional requirements, and personal health constraints, Prasetyo and Baizal [60] employed SWRL rules in the ontology-based system design. This system deduced suitable meal recommendations based on advanced reasoning, e.g., high-cholesterol food avoidance in individuals with cardiovascular risk or fiber increase for individuals with constipation risk. Through the use of ontology, the study provided evidence-based, traceable, and explainable recommendations, which are particularly beneficial among the elderly. Research findings from this study demonstrated the potential of ontologies in delivering a schema for semantic reasoning in nutrition systems to enable personalized and context-sensitive dietary care for older adults.

4. Human–Computer Interaction (HCI) and Personalization Models: A number of papers addressed the user experience through HCI models, Participatory Design, and Ageing-Centered Design Principles, emphasizing inclusivity and usability in design for elderly populations. For example, Weber et al. [61] employed a Participatory Design (PD) approach to develop diet-support AI technologies that directly addressed the needs of older populations. PD is centered on direct end-user involvement at each phase of the design process to ensure that technology interventions are aligned with their experienced realities, values, and interests. The approach is particularly applicable in older populations, where usability, emotional comfort, and accessibility are most critical for long-term adoption. By employing the PD strategy, Weber et al. [61] developed an easy-to-use digital nutrition assistant that integrated personal reminders, speech assistance, and easy-to-use interfaces. This article highlights how PD is not merely making interventions more usable but also stimulating feelings of ownership and control among older adult users, making resulting interventions more sustainable and effective. It also placed a strong focus on requiring empathic design practices that go beyond technical functionality to include the social and psychological processes of eating in later life.

Chao and Hass [54] applied the Ageing-Centered Design Principles (Simplicity and Cognitive Load Reduction, High Visibility and Legibility, Predictability and Familiarity of Interaction Models, Emotional Sensitivity and Respect for Autonomy, and Pacing and Temporal Flexibility) to design a diet-support AI system that is tailored to older adults, and further optimized for usability, comfort, and independence. The layout reduced cognitive load through simplified interfaces and step-by-step sequential interaction, and visual accessibility was enhanced through the use of large fonts, high-contrast color schemes, and familiar icon sets. Predictability and shared interaction metaphors minimized learning barriers, and the system was emotionally sensitive through the inclusion of dietary suggestions as soft hints rather than instructions. Flexible timing and self-motivated engagement were also supported because older adults prefer non-pressured interaction. By basing these guidelines, the intervention built trust, reduced frustration, and facilitated control of their own nutrition by the older adults.

5. Optimization and Control Models: A subset of the reviewed papers showed a number of approaches such as Genetic Algorithms (GAs), Fuzzy PID control, and Multi-Constraint Particle Swarm Optimization to model personalized intervention strategies or optimize interaction timing and delivery. For example, Parikh et al. [62] integrated Genetic Algorithms (GAs) aimed at optimizing personalized meal planning and dietary control into a nutrition-based system for older adults. Genetic Algorithms simulate natural selection by iteratively evolving solutions toward optimal configurations. These algorithms are particularly useful for solving complex, multi-variable problems where traditional optimization techniques may struggle. GAs were used to create an intelligent system that adapted meal suggestions based on physiological inputs, user preferences, and dietary restrictions, in combination with Fractional Order PID (FOPID) controllers, Forward and Inverse Kinematics, and Fuzzy PID logic. The GA component helps minimize the risks of different health conditions, such as high blood pressure, diabetes, and cholesterol sensitivity, common in aging populations, while searching for optimal food combinations that meet nutritional targets.

This algorithm evaluated multiple candidate meal plans by using a fitness function that balanced nutritional adequacy (e.g., protein, fiber, and micronutrients), palatability, and compliance with medical constraints. Following successive iterations, the final system provided suitable options while refining its suggestions for individualized health support. This approach enabled dynamic adjustments, where the system could re-optimize recommendations in response to changes in user feedback or medical status. By employing Genetic Algorithms, the study demonstrated how adaptive, data-driven optimization can enhance the personalization and effectiveness of dietary interventions for older adults. This method provided a scalable and intelligent mechanism to balance competing dietary demands, thereby reducing decision fatigue and increasing adherence to healthy eating patterns.

#### 4.1.2. Distribution of Eating Patterns Tracked Using AI Technologies Among Older Adults

We employed descriptive statistics to count the occurrences of articles based on the different eating patterns of older adults. Our analysis shows a particular emphasis on monitoring structured meals (breakfast, lunch, dinner) and related behaviors to inform personalized nutrition and health interventions. Table 1 summarizes our findings. Our results show that breakfast, lunch, and dinner were explicitly mentioned in most of these studies, indicating that AI systems are designed to capture full-day meal cycles. Breakfast appeared in datasets and models either through meal planning, food logging, or as part of food recommendation systems in at least 16 distinct entries, while lunch and dinner were equally well represented—often together—across meal tracking, delivery, and behavioral monitoring studies.

Several studies included snack tracking as well, highlighting attention to in-between-meal consumption. For example, Nolting and Wittke [63] captured and analyzed food logging, including breakfast, lunch, dinner, and snacks, using a digital coaching platform supported by fuzzy logic. The digital coaching platform provided users with adaptive feedback for diabetes prevention. Similarly, Gautam and Gulhane [64] employed an optimization algorithm to automatically generate daily meal plans, explicitly including snacks, based on user profiles and nutritional requirements. In addition, Chifu et al. [65] leveraged a bio-inspired computational model to produce full-day dietary schedules that incorporated morning and afternoon snacks alongside main meals, ensuring balanced nutrition across eating occasions.

In addition, we found other eating patterns related to tracking of meal behavior through smart systems, such as gesture-based detection, e.g., hand-to-mouth actions [66], image-based assessments [67], motion/contact sensors [68], and robot-assisted feeding [62], especially in contexts requiring support for older adults with physical or cognitive limitations. These methods provided not only confirmation of food intake but also enabled monitoring of eating behavior quality, such as bite intervals, meal frequency, and meal completeness. For example, Ioakeimidis et al. [69] applied automated bite detection algorithms to capture mouthful intervals and bite-to-bite timing, enabling objective measurement of eating pace and potential motor impairments. Similarly, Konstantakopoulos et al. [67] classified and quantified food items from user-taken images using AI-powered image recognition to support detailed analysis of meal composition and frequency. Additionally, Derouiche et al. [68] deployed motion and contact sensors to detect when meals were consumed, allowing for unobtrusive monitoring of meal frequency and completeness in real-world settings.

Daily and weekly personalized meal plans, generated through AI, commonly consist of all three meals that are frequently aligned with dietary guidelines or therapeutic diets, including the Mediterranean diet [67]. For example, Xu et al. [59] recommended three daily meals, aligning with traditional Chinese dietary practices and tailored to individual health conditions, using a knowledge-based AI framework. Similarly, Gautam and Gulhane [64] generated daily meal plans, incorporating breakfast, lunch, snacks, and dinner that meet nutritional and cultural requirements through the application of an optimization algorithm. In addition, Prasetyo and Baizal [60] leveraged semantic reasoning to create daily meal plans—breakfast, lunch, and dinner—aligned with users’ health profiles and nutritional guidelines.

In addition to meal tracking on a physical basis, behavior and context were also included. These involved the examination of eating habits, for example, missing meals and food choice [51], tracking nutrient consumption, such as macronutrient/micronutrient intake [57], and food interaction, such as recipe searching, food recording, and chat conversation promoting healthy food choices [61]. For example, Jo et al. [51] received participants’ self-reported eating behaviors, including meal skipping and meal quality, through conversational AI-based personalized public health feedback. Similarly, Patino-Alonso et al. [57] analyzed comprehensive data on nutrient intake in an attempt to calculate dietary adequacy and estimate risk of early vascular aging. In addition, Weber et al. [61] engaged users in conversational interviews, which allowed them to log what they had eaten, request recipes, and track nutritional intake using a participatory-designed chatbot. Additional studies emphasized mealtimes as a family and social occasion, capturing the planning, shopping, preparation, eating, and clean-up phases, thereby collecting thorough information on the mealtime routines of older people. For example, Wu et al. [53] explored family meal coordination as an interdisciplinary process, from food planning and preparing to eating together and after-meal cleaning, illuminating the social and logistical foundation of mealtime at home.

Overall, AI technologies in aging-related nutrition research and applications can increasingly capture multi-layered food consumption activities, from isolated meal events to overall lifestyle habits, to lay strong foundations for personalized, adaptive, and context-sensitive nutrition support systems for older adults.

**Table 1:** Summary of the different eating patterns of older adults tracked using AI technologies.

S/N	Reference	Eating Patterns	Performance Metrics
1.	[70]	Breakfast, lunch, dinner selections and personalized eating preferences considered	Accuracy: 63.1% (fusion model), User satisfaction ~60%
2.	[69]	Mouthful intervals, bite-to-bite analysis	Correlation R > 0.95 with manual annotations
3.	[53]	Family meal processes including breakfast, lunch, and dinner coordination	No formal performance metrics (qualitative study)
4.	[51]	Meal intake patterns, Health-related meal behaviors	Improved disclosure with LLM chatbot; qualitative engagement outcomes
5.	[63]	Meal-based food logging (breakfast, lunch, dinner, snacks)	Average weight loss: 5.8 kg (treatment) vs. 3.5 kg (control)
6.	[71]	Three-course meals (appetizer, main dish, dessert)	Recall@20 = 0.3753 (CrossCBR best model)
7.	[72]	Meal preparation tracked (as high-level daily activity)	Weighted F1 > 90% (semi-supervised HAR with sparse labels)
8.	[73]	Monitored through personalized nutrition planning	No empirical metrics (conceptual framework)
9.	[74]	Adapted based on seasonal changes and health needs	Emotion recognition accuracy: 84%
10.	[75]	Daily meal decisions and healthy food recognition supported	80% preferred voice assistant over DSM apps; usability ratings
11.	[76]	Monitored through dietary intake tracking and recommendations	Random Forest models promising; no numeric accuracy reported
12.	[77]	Healthy eating promotion integrated into dialogue	Qualitative outcomes; small pilot (no numeric metrics)
13.	[66]	Eating behavior analyzed by gesture intervals (hand-to-mouth actions)	Segmental F1 = 0.944 (MS-TCN, IoU = 0.5)
14.	[67]	Based on Mediterranean diet food categories (breakfast, lunch, dinner patterns)	Top-1 Accuracy = 82.4%, Top-5 Accuracy = 97.5%, MAPE = 10.7%
15.	[78]	Meal completeness, intake variability, dietary behavior patterns analyzed	Accuracy = 94%, Recall = 92% (RF, AdaBoost, Logistic Regression)
16.	[60]	Breakfast, lunch, dinner recommendations based on personal health profile	Precision = 87.5%, Recall = 100%, F1 = 93.3%
17.	[68]	Detected using unsupervised clustering, based on motion and contact sensor data	Davies–Bouldin Index (K-Means best)
18.	[62]	Not explicitly detailed as eating patterns but focused on enabling self-feeding tasks	Positional error: 0.67% (overshoot, GA-FOPID controller)
19.	[61]	Monitored through food diary and nutrition logging features	Moderately effective; usability insights (no numeric accuracy)
20.	[57]	Not explicitly discussed (focus on nutrient intake)	Accuracy = 68.8%, Sensitivity = 66.7%, Specificity = 68.8% (stacked ensemble)
21.	[59]	Three meals per day recommendations aligned with traditional Chinese meal structure	Improved diet quality & diversity (*p* < 0.001, simulation study)
22.	[58]	Not directly studied	Accuracy = 95.07% (SVM for dysphagia screening)
23.	[56]	Daily meal logging, food categories based on dietary habits	Usability: SUS = 85.83, Object recognition rate = 91% (with hybrid mode)
24.	[64]	Daily meal plan creation with food categories	Fitness ≈ 0.9999 after ~30 iterations (PSO algorithm)
25.	[79]	Not discussed explicitly; focus on food product nutrition information	Qualitative usability; small sample, no numeric metrics
26.	[54]	Breakfast meal selection based on diabetes-appropriate food recommendation	SUS, NASA-TLX; Nutri-score format reduced workload
27.	[65]	Full-day meal pattern (breakfast, morning snack, lunch, afternoon snack, dinner)	Fitness > 0.90; execution time reduced by ~80–90% (hybrid immune algorithm)
28.	[80]	Daily meal patterns analyzed (energy, macro and micronutrient intake)	High agreement with expert nutritionists (qualitative validation)
29.	[81]	Daily and weekly eating pattern monitoring for nutritional adequacy	Qualitative evaluation; prototype stage, no numeric metrics
30.	[82]	Focused on nocturnal glucose fluctuations rather than eating patterns	Accuracy ≈ 90% (CNN + CDAE for hypoglycemia detection)

### 4.2. How Have the Different AI Technologies Been Used to Promote Healthy Eating Habits Among Older Adults? (RQ2)

**Figure 2:**
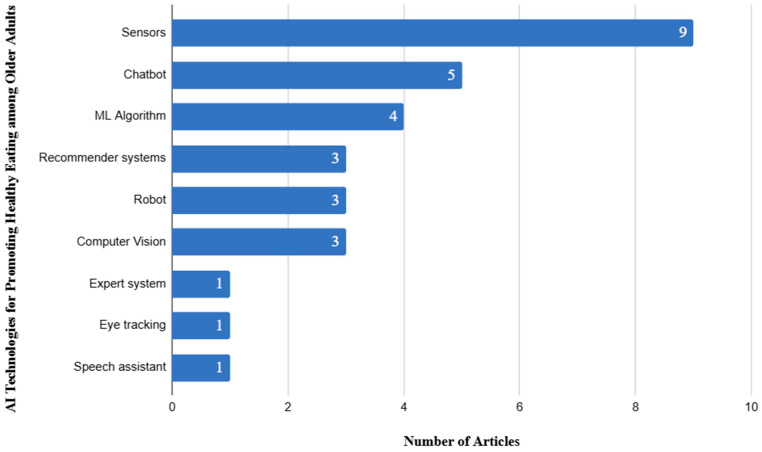
Distribution of AI-technologies employed in articles we reviewed.

To answer this question, we employed descriptive statistics to count the number of occurrences of articles based on the different AI technologies that have been explored for promoting healthy eating habits among older adults. Furthermore, we expanded their applications. Based on our analysis, we found diverse collections of AI technologies employed to promote healthy eating habits among older adults (Figure 2). Among the commonly used technologies employed in studies we reviewed, sensor-based technologies appeared in ten studies (e.g., [66,67]). These sensors, often embedded in wearables or integrated into home-based systems, enabled the detection of eating-related gestures, such as hand-to-mouth movements, and monitored physiological indicators to assess meal timing, frequency, and nutritional intake. For example, Nolting and Wittke [63] tracked weight, users’ physical activities, and food logs through the combination of GSM-based body scales and fitness bands. This resulted in a 41% rate of clinically significant weight loss in the treatment group. Similarly, Wang et al. [66] employed wrist- and head-mounted accelerometers and gyroscopes to segment eating gestures through a detailed analysis of bite intervals and meal patterns. This achieved an F1-score of 0.944.

Chatbots, which were used as conversational agents in delivering personalized nutrition guidance (e.g., [60,61]), were among the widely used in studies we reviewed, featuring in five studies. These agents provided interactive support for meal planning, answered diet-related questions, and offered emotional encouragement, making dietary advice more accessible and engaging, particularly for older adults living alone or in resource-constrained settings. For instance, Fridolin allowed users to log meals, request recipes, and receive proactive reminders, which improved engagement during the initial two weeks of use [61]. In another related study, ontology-based healthy food recommendation used semantic reasoning to deliver meal suggestions based on individual health profiles, achieving high accuracy compared to nutritionist-validated recommendations (F-score = 0.933). Machine learning algorithms, which were applied to analyze eating behaviors, predict dietary risks, and tailor nutritional interventions based on individual health profiles such as glucose levels, weight trends, or micronutrient deficiencies, appeared in four studies. For example, Di Martino et al. [78] combined dietary intake data with bioimpedance-based body composition measures, achieving over 94% accuracy in malnutrition risk detection. Similarly, Patino-Alonso [57] applied a stacked ensemble model to dietary nutrient data, identifying key dietary risk factors such as sodium, protein, and calcium intake.

Recommender systems, which generate meal plans and food suggestions, aligning with personal preferences, cultural contexts, and medical conditions, were employed in four studies (e.g., [59,64]). Xu et al. [59], through the tailoring of meal combinations to individual health conditions such as hypertension or diabetes, improved diet quality and diversity significantly (*p* < 0.001). Similarly, Gautam and Gulhane [51] generated optimized daily meal plans, including snacks, based on nutritional requirements, cultural food preferences, and disease-specific dietary restrictions. Robotic technologies, used in three studies, focused on feeding assistance and interactive dietary support. For instance, Parikh et al. [62] tracked users with severe motor impairments who were allowed to eat independently using a robot. This attained a high accuracy in spoon positioning and minimal overshoot. In addition, Yao et al. [74] integrated the Pepper robot to provide seasonal dietary advice alongside emotional support, reducing feelings of isolation while promoting healthier food choices.

Computer vision technologies, which supported image-based food logging and dietary assessment, were employed in three studies. Konstantakopoulos et al. [67] used smartphone-based image recognition to classify Mediterranean diet items with a top-1 accuracy of 82.4%, supporting portion estimation for glucose control. Also, Elfert et al. [56] combined object detection with manual verification to log meals for frail older adults, achieving a 91% recognition rate and high usability scores (SUS = 85.83).

Less frequently used but equally innovative technologies included expert systems, eye-tracking, and speech assistants, each appearing in one study. Cioara et al. [80] provided rule-based dietary interventions matching expert nutritionists’ assessments for malnutrition detection and dietary planning. Chao and Hass [54] incorporated eye-tracking to assess attention to nutrition labels, finding that Nutri-Score formats reduced cognitive workload. Similarly, Seiderer et al. [79] provided nutrient information for German Seniors by enabling barcode scanning and voice-based food information retrieval, with seniors valuing its privacy-first design.

Based on our review findings, we found that, while sensor technologies dominate the field, the integration of chatbots, machine learning, recommender systems, computer vision, and assistive robotics reflects a growing commitment to developing AI systems that are not only intelligent but also inclusive, responsive, and supportive of older adults’ unique dietary and health needs.

### 4.3. How Effective Are AI Technologies for Promoting Healthy Eating Habits Among Older Adults? (RQ3)

We employed descriptive statistics to count the number of articles that have reported the effectiveness of AI interventions in supporting healthy eating behaviors among older adults. We classified the effectiveness levels into effective (for cases where articles reported a good level of accuracy in AI models and interventions), moderately effective (for studies that reportedly show an average performance of the AI intervention), and partially effective (for studies with below-average performance and potential for improving this performance). Overall, we based the performance of these interventions on the accuracy of the intervention, user satisfaction, and measurable health benefits, which shows their potential applicability in both experimental and real-world settings. We found that most AI models reported in the studies we reviewed achieved good performance metrics. For instance, ensemble models like AdXGRA achieved up to 68.8% accuracy [57], while advanced machine learning (ML) classifiers yielded results as high as 94% accuracy and 92% recall [78]. Additionally, the models for image-based food identification and estimation of food volume also attained top-1 accuracy of 82.4% and mean absolute percentage error (MAPE) of 10.7% [67], indicating high dietary monitoring accuracy. We also found that emotion-aware systems and gesture-based monitoring frameworks attained over 0.90 F1-scores [66], thereby proving their performance in interpreting and adapting to user behaviors.

Beyond the accuracy of interventions employed, several systems in studies we reviewed demonstrated real-world health outcomes. For example, one of these interventions reported an average weight loss of 5.8 kg in the treatment group, with 41% achieving more than 5% body weight reduction [63]. In other contexts, such as improvements in diet quality, dietary diversity, and food-related decision making (*p* < 0.001), some of these interventions showed better outcomes of AI on nutritional health [59]. Assistive technologies such as robot-fed systems and dysphagia detection tools, which directly support users with feeding challenges [58,62], achieved up to 95% detection accuracy.

Other key indicators showing the effectiveness of these interventions were usability and user experience. For example, one of the studies employed Google Home for diet self-management and found that these were preferred by 80% of users over mobile apps, enhancing satisfaction and ease of use [75]. Similarly, voice-interactive systems were also rated by older adults highly in terms of privacy facilitation and presentation style clarity [79] of nutritional label styles, for instance, Nutri-score reduced cognitive workload considerably and improved comprehension [54]. While most interventions were piloted and validated through user studies or experimental testing, some were still in prototype or conceptual phases pending further deployment and testing. These included integration frameworks for anti-aging care [73] and socially aware eating environments [53].

**Figure 3:**
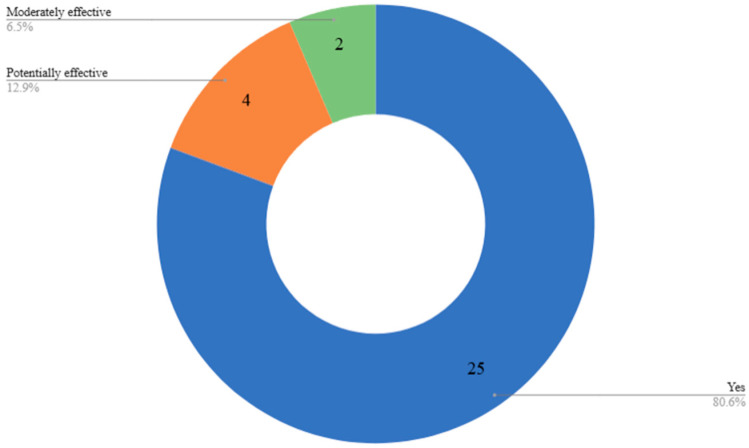
Distribution of AI-technologies employed in articles we reviewed.

Overall, most of these AI-enabled systems (n = 25, 80.6%) were found to be effective in promoting healthy eating habits among older adults, a smaller portion (n = 4, 12.9%) showed potential effectiveness, indicating promising conceptual or preliminary outcomes, while only n = 2, 6.5% were categorized as moderately effective, often due to limited adoption or early-stage development (review dataset)—(Figure 3). Table 2 presents a summary of the performance of the different AI technologies employed in the articles we reviewed.

### 4.4. What Are the Limitations and Ethical Implications of Designing AI Technologies for Promoting Healthy Eating Habits Among Older Adults? (RQ4)

We conducted our thematic analysis by following Braun and Clarke’s six-step framework [48]: (1) familiarization with the data, (2) generating initial codes, (3) searching for themes, (4) reviewing themes, (5) defining and naming themes, and (6) producing the report. Two researchers independently coded all extracted limitation statements from the included studies using NVivo 14. Interrater reliability was assessed, which yielded an overall agreement of 89% and Cohen’s κ = 0.78, which indicates substantial agreement [83]. Any discrepancies were resolved through discussion until consensus, and the reconciled dataset was synthesized into overarching themes. Our analysis shows several limitations of AI interventions designed to support healthy eating among older adults. Through iterative comparison and theme refinement, these were merged into five overarching themes: prototype and laboratory-centric evaluation, sample size and study design limitations, technology and measurement gaps, equity, diversity, and participation bias, ethical, privacy, and clinical relevance concerns. One general limitation was the lack of in vivo implementation and excessive reliance on prototype or simulation phases. A majority of the interventions were tested only in laboratory environments, with no clinical or large-scale field tests [73,81], hence compromising their generalizability. Over 10 of the studies we reviewed reported fewer than 10 to 30 participants [75,79], reducing statistical power and the ability to detect relevant effects in representative populations. Other common issues were the use of artificial datasets or experimental settings, e.g., simulated meals, pre-defined cycle lengths, or small numbers of food categories, that failed to represent real-world variability in eating behavior [57,62,71]. In addition to these, we found that multiple systems were not coupled with real-time sensors or health tracking, making them incapable of offering continuous, adaptive feedback [60], and some did not track real food items or quantities, using gesture-based inference instead, which may not accurately represent dietary intake [66].

Ethical and equity-related limitations also emerged. A few studies highlighted limited demographic diversity, such as being restricted to predominantly Western or younger users [53,56], raising concerns about the inclusivity and fairness of AI models when applied to frail or ethnically diverse elderly populations. Additionally, the use of self-selected samples, compensation-related bias, and high dropout rates introduces sampling bias and raises questions about user motivation and the authenticity of engagement in these studies [63,78]. Some studies lacked experimental control or had short durations, limiting the ability to observe long-term dietary behavior change or health outcomes [51,72]. Others did not control confounding factors (e.g., medication use, comorbidities), particularly in cross-sectional designs [57], making it difficult to attribute outcomes solely to the AI interventions.

From an ethical standpoint, the limited testing in free-living, real-world conditions raises concerns about user autonomy, safety, and reliability. Many systems remain under-evaluated in vulnerable elderly populations, which may lead to unforeseen consequences if deployed prematurely. Furthermore, data privacy, transparency of algorithmic decisions, and the risk of over-reliance on AI in personal health management must be critically considered before widespread implementation. In summary, while AI technologies for promoting healthy eating among older adults have shown numerous potentials, current research is constrained by small samples, limited deployment, prototype-level functionality, and insufficient attention to demographic inclusivity and ethical design. To safeguard sensitive health information, AI developers should implement systems with advanced privacy-preserving techniques. Additionally, informed consent processes should be user-friendly and easy to understand. Future efforts must prioritize rigorous, real-world validation, larger and more diverse samples, and ethically grounded design principles to ensure these systems are safe, equitable, and truly beneficial to aging populations. Collaboration with healthcare professionals, including registered nutritionists, geriatricians, and speech pathologists, is vital to the AI development process. Their insights help guarantee that AI-derived dietary recommendations are clinically relevant, prioritizing the safety of older users and addressing specific conditions such as dysphagia (difficulty swallowing) or polypharmacy (the concurrent use of multiple medications). This collaborative effort should be maintained throughout all stages of the AI tool’s lifecycle, including design, testing, and deployment, to ensure the recommendations are practical and impactful.

### 4.5. What Are the Future Implications of Designing AI Technologies as Nutrition-Based Interventions for Older Adults in Future Research? (RQ5)

We analyzed recommendations for future AI-driven nutrition interventions using Braun and Clarke’s six-step thematic analysis framework [48]. Two researchers independently coded all extracted recommendation statements from the included studies using NVivo 14. Interrater reliability between the two coders showed 88% overall agreement with a Cohen’s κ = 0.78, reflecting substantial agreement [83]. Coding discrepancies were discussed and resolved by consensus, and the finalized dataset was synthesized into six overarching themes: real-world deployment and validation, integration with wearables, sensors, and health systems, inclusive and adaptive design, ethics, privacy, and trust, advanced AI and holistic functionality, interdisciplinary and clinical collaboration. The findings reveal a trend across studies on the need to advance AI-driven nutrition interventions from prototype to real-world application, with a focus on improving adaptability, inclusiveness, and effectiveness for older adult populations. The main future aim of these studies was tailored toward real-world deployment and empirical validation [59,65]. Many studies emphasized the importance of field-testing AI systems in free-living environments, moving beyond lab or simulated settings to understand usability, acceptability, and long-term behavioral impact [66,67]. Recommendations included larger sample sizes, inclusion of diverse populations (e.g., diabetics, prediabetics) [63], and longer-term tracking of health behaviors such as diet, physical activity, and sleep [73,78], to better reflect the complex realities of aging and nutrition management.

Integration with wearable devices, smart home systems, and assistive technologies was highlighted as a key opportunity to enable continuous, multimodal data collection [72,76]. Future designs are encouraged to leverage sensor data, real-time nutrient monitoring, and adaptive feedback systems to offer highly personalized and context-aware recommendations [67,78]. This includes connecting AI models to electronic health records (EHRs) for more accurate prediction and guidance based on individual health profiles [59]. Improving accessibility and inclusivity is another major implication. Future systems should support multi-modal input (e.g., voice, gesture, touch), offer adjustable narration speeds, and account for sensory impairments such as hearing loss or vision limitations [75,79]. There is also a need to develop domain-specific speech recognition and expand AI vocabulary to accommodate elderly users’ interaction styles [54].

Several studies emphasized the importance of privacy protection, ethical considerations, and ease of installation to enhance user trust and system adoption [77,79]. Proposed directions include simplified onboarding, proactive yet balanced interactions (e.g., through playful features like riddles) [61], and ensuring robust data governance in integrated platforms. From a technological perspective, recommendations included incorporating hybrid interaction models, cooperative AI behavior, and health-aware recommendation algorithms that go beyond personalizing preferences to actively encourage healthier choices [71]. Expanding the scope of food databases, object detection (e.g., with depth sensors), and activity modules, including exercise, stress, and sleep tracking, was also seen as crucial for holistic interventions [56,63]. Finally, researchers have emphasized the need for interdisciplinary collaboration, particularly with healthcare professionals, to align AI nutrition tools with clinical practices and standards [80]. Many studies suggested integrating these technologies into telehealth platforms or community-based care models to reach older adults more effectively and equitably [73].

In summary, the future of AI nutrition-based interventions for older adults will require real-world implementation, multimodal sensing, adaptive and inclusive design, and clinically informed development, all driven by rigorous evaluation to ensure long-term effectiveness and ethical integrity.

## 5. Discussion

This section presents discussion on key results from this review, suggesting the growing body of research on AI technologies designed to promote healthy eating among older adults, synthesizing trends in publication, methodological approaches, and intervention effectiveness. We highlight the promising technical capabilities, emphasizing the importance of rigorous clinical evaluations and comprehensive nutritional risk management, including safety concerns like choking and dysphagia. Overall, the discussion provides future directions for designing impactful, equitable, transparent, and user-centered AI systems for aging populations.

### 5.1. Trends in the Application of AI Intervention for Promoting Healthy Eating Among Older Adults and Their Overall Effectiveness?

Overall, our review demonstrates that AI-driven interventions tailored to support healthy eating among older adults have been shown to be effective. This effectiveness covers technical accuracy, user acceptance, measurable health outcomes, and behavioral engagement, consistent with international systematic reviews reporting positive impacts of AI and digital health tools on diet quality, nutritional status, and clinical indicators in aging populations [41,84,85,86]. The effectiveness of these diverse AI models, from ensemble machine learning classifiers achieving accuracy up to 94% for food image recognition systems [78] and voice-interactive chatbots [51,60], confirms the practicality of integrating AI applications in both controlled and real-world contexts [41,85]. This is important given that this review has shown the need to help older adults make personalized dietary choices based on the different underlying conditions, including age-related chronic illnesses (hypertension, coronary heart disease, stroke, accelerated vascular aging, multi-morbidity), metabolic disorders (prediabetes, newly diagnosed or long-standing type 1 and type 2 diabetes, dyslipidemia, hypoglycemia), neuro-degenerative and neurological challenges (Parkinson’s disease, mild cognitive impairment, post-stroke disability, hand amputation, dysphagia), geriatric syndromes (frailty, dehydration risk, malnutrition, nutrient deficiencies), sensory and functional decline (vision or hearing loss, technological unfamiliarity), and psychosocial factors that compound health risks (social isolation, loneliness, depression, “lonely death” risk). By considering how dietary interventions promote healthy eating, we demonstrate how they can mitigate further chronic-disease progression and improve overall well-being in later life.

Based on our bibliometric trends, especially the increase in the number of publications since 2020, this reflects a growing scholarly and practical interest of the research communities in tailoring technological interventions for the aging population [86,87]. In relation to their effectiveness in providing support for this population, we found that a wide range of AI technologies consistently delivered measurable benefits for diet, health monitoring, and caregiving: fusion-based algorithms surpassed competing methods with 63% accuracy while still earning ≥ 60% user satisfaction [70]; cooperative and ensemble recommenders attained a good level of accuracy (AdXGRA ≈ 69%) [57], K-Means lowest Davies–Bouldin Index (DBI) [68], and up to 94% accuracy/92% recall in specific models [78]) and improved diet quality, diversity, and user engagement, especially in the first 5–10 days when proactive messaging was used. Sensor-centric systems matched nutritionists’ interventions, detected dysphagia with 95% accuracy [58], flagged nocturnal hypoglycemia at ~90% [82], and maintained high agreement (R > 0.95) with manual bite counts [69], while emotion-aware agents achieved 84% recognition and real-world success in daily coaching [74]. Voice-based platforms proved popular (80% preferring Google Home over mobile apps) [75] and usable (SUS ≈ 86) [56], with seniors valuing privacy-preserving designs. Robotics and adaptive controllers provided safe feeding assistance (0.67% overshoot) [62] and achieved precise food-volume estimation (MAPE ≈ 10.7%) [67], and anti-aging frameworks [73], GA-FOPID optimizers, and CLONALG variants all reached fitness values > 0.90 with markedly reduced runtimes [63]. Collectively, these results show that well-integrated AI technologies, covering fusion analytics, machine-learning-driven recommendation, multimodal sensing, and conversational or robotic interfaces, not only outperform traditional baselines but also provide tangible clinical, nutritional, and usability gains for the target population. These findings align with existing work showing the potential of these AI technologies [36,86]. This implies that, unlike more aggregated prior reviews, we highlight the differential success of varied AI technologies.

However, despite AI’s effectiveness in delivering healthy eating interventions, we found contrasting results on the persistent challenges, adoption barriers related to digital literacy, technological accessibility, and cultural relevance, limiting the broader generalization and sustained engagement [86,87,88]. For example, geographic concentration of research in Asia and North America illustrates ongoing disparities in inclusivity and contextual applicability. To overcome these gaps, future efforts must prioritize field deployments that integrate user-centered and participatory design, consider cognitive and sensory variability among older adults, and embed theory-driven behavioral strategies to promote long-term adherence such as [85,86,87].

In summary, the synthesized evidence from bibliometric patterns and verified effectiveness outcomes positions AI-driven nutritional interventions at the forefront of innovation. These interventions offer scalable, personalized, and adaptive solutions with proven benefits but require further expansion into diverse real-world settings, inclusive design frameworks, and rigorous behavioral incorporation. Addressing these priority benefits will be crucial to sustained global nutritional health improvements and enhanced quality of life for aging populations worldwide [41,84].

### 5.2. Challenges and Implications of Tailoring Interventions for Older Adults in the Real World: Clinical Versus Home Settings

Recent advances in AI technologies for promoting healthy eating among older adults have revealed both exciting opportunities and significant challenges when it comes to deploying these interventions beyond controlled environments into real-world clinical and home settings. A recurring limitation in literature is the predominance of prototype or simulation-phase studies with minimal clinical validation and few large-scale field deployments, restricting the generalizability and practical impact of reported successes. This has also become evident in existing work. For example, a narrative review conducted by Abrahams and Raimundo [89] revealed persistent industry challenges, including difficulties scaling interventions for real-world use. Compared to research settings (controlled environments), interventions often benefit from structured oversight, access to health records, and professional support when they are deployed in clinical settings, facilitating rigorous monitoring and adjustment of dietary recommendations. However, these controlled environments may not fully capture the everyday variability in older adults’ eating behaviors, comorbidities, medication schedules, and psychosocial dynamics, nor do they reflect real-life barriers to technology use such as sensory impairment, cognitive decline, or digital literacy gaps.

Additionally, transitioning to home settings, where interventions have the potential of supporting independent living, introduces unique complexities. Results from our review show small sample sizes, use of simulated data, and short study durations, all of which limit the capacity to reliably assess long-term behavioral change and measure the effects of AI interventions as they interact with the nuanced realities of aging at home. In-home implementations must contend with highly variable self-management capabilities, changing routines, and a wide spectrum of health conditions. For interventions to be truly effective, they must integrate seamlessly with wearables, smart appliances, and everyday objects to enable continuous, multimodal data collection and adaptive feedback. Yet, several systems still lack real-time sensor integration or depend on gesture-based proxies for eating, potentially missing crucial aspects of dietary intake or failing to respond dynamically to shifting health states.

Aside from the gap in clinical application of AI interventions tailored for promoting healthy eating behaviors among older adults, our review shows a significant gap in the eating patterns they are tailored to support. Studies we reviewed show that current AI nutrition interventions for older adults rarely address risks such as choking and swallowing safety (dysphagia), despite their clinical importance for this population. We found that most interventions emphasize routine dietary tracking—including breakfast, lunch, and dinner selection [59,60,70], personalized meal planning [64,65,81], mouthful interval and bite analysis [63,66,68,69], meal-based logging [56,61,67,78], and behavioral or contextual monitoring [51,53,72,74]. However, the acute health risks of choking, aspiration, and related swallowing disorders are largely understudied from the design, evaluation, and reporting of these technologies. This is important because recent clinical reviews affirm that dysphagia affects 10–33% of the elderly and carries substantial risks for choking, aspiration pneumonia, dehydration, and even mortality [90,91,92]. Strategies for safe food texture modification and tailored diet planning to reduce these risks, such as gelation, nonthermal processing, and 3D food printing, are a focus of nutrition science but rarely integrated into the AI models or datasets used in reviewed interventions. Few AI systems in our survey incorporate real-time sensor monitoring of swallowing or explicitly connect dietary recommendations to safety metrics for at-risk older adults, reflecting a narrow focus on intake and behavior rather than on critical safety concerns. In contrast to our findings, some recent development efforts outside our core review have begun exploring ontology-based decision support for dysphagia and safe swallowing (for example, targeting neuromuscular patients), and clinical guidelines stress the importance of regular safety monitoring and personalized intervention protocols for residents in care settings [90,91,93].

Overall, our findings suggest the implications for designing impactful AI nutrition interventions with emphasis on moving toward rigorous real-world validation, with larger, more heterogeneous samples tested over extended periods in both clinical and home environments. Hence, future interventions should prioritize multimodal and accessible input tailored for age-related changes, offer context-aware personalization by leveraging electronic health records and sensor data, and uphold strong privacy and ethical standards to build trust and adoption. Collaboration with healthcare professionals and integration into telehealth and community care networks will be crucial to ensure clinical relevance while supporting independent self-management at home. Ultimately, bridging the divide between prototype efficacy and sustained, real-world impact will require an interdisciplinary, user-centered approach, one that meets the diverse needs and safeguards the well-being of older adults wherever they live. In addition, based on current advances in AI intervention, there will be a need to move beyond general dietary management and explore multidisciplinary approaches that can detect and respond to choking risks, support texture-modified diets, and deliver recommendations that are both nutritionally sound and clinically safe for frail and high-risk older adults [90,91,93]. Addressing this gap will require integrating swallowing risk assessment, personalized texture and fluid management, and continuous sensor-based safety feedback into real-world AI applications, priorities that are increasingly recognized in geriatric nutrition and interdisciplinary aging research [90,91,93].

### 5.3. Ethical Considerations for Deploying AI Interventions in the Future

Building on our proposed requirements for advancing the future of AI-driven nutrition interventions from prototype phases toward robust clinical evaluations and inclusive nutritional tracking, including addressing pivotal risks such as choking and dysphagia, we present ethical considerations that will guide responsible development and deployment. As AI tools continue to gain research attention in supporting older adults’ dietary health, ensuring their safety, equity, transparency, and respect for user autonomy becomes important to translating technological potential into meaningful and trustworthy real-world impact.

Our review aligns with recent scholarly perspectives emphasizing that ethical deployment requires AI systems trained on diverse, evidence-based datasets to avoid bias and support inclusivity, particularly given that older adults vary widely in cultural, cognitive, and physiological characteristics [89]. Transparency and explainability are essential to prevent “black box” effects where users and clinicians cannot understand or trust AI recommendations, which is critical when dietary advice interacts with complex health needs [89,94]. Ethical frameworks will also require embedding user autonomy by ensuring individuals can control and contextualize AI-driven recommendations, as well as clear communication about data use and privacy safeguards, especially when sensitive health data and real-time monitoring are involved [89]. Further, the integration of behavior change theories within AI designs must be transparent and ethically sound to build trust and avoid manipulative practices [89].

Importantly, current AI nutrition tools largely overlook pressing safety risks like choking and dysphagia, which carry severe consequences for frail older adults. Ethical deployment mandates addressing these gaps through clinical testing, inclusion of swallowing risk assessment, and integration of sensor-based real-time safety monitoring (e.g., for aspiration events) as part of personalized nutrition solutions [89]. Ensuring interventions do not harm vulnerable users through incomplete or inaccurate guidance must be a priority, reflecting principles of do-no-harm and beneficence fundamental to healthcare ethics [94,95]. This is especially important as many existing studies remain confined to controlled or simulated environments, lacking robust evidence from free-living, diverse cohorts where real-world risks are pronounced. Moreover, barriers such as digital literacy disparities, high costs, and infrastructure limitations raise concerns about fairness and equity, risking exclusion of socially vulnerable and ethnically diverse older adults from the benefits of AI nutrition support [88]. Ethical frameworks emphasize the need for inclusive design, multimodal and accessible interfaces (voice, gesture, touch), and participatory approaches that involve end users and caregivers in co-creating trustworthy systems [89]. Regulatory compliance, such as adherence to AI safety standards, is also critical to safeguard user rights and build confidence in technology [89,96]. In summary, the ethical considerations for future AI nutrition interventions require a multifaceted approach in combining rigorous clinical validation that includes safety monitoring for choking and swallowing, equitable access and inclusivity, transparency and autonomy in data and decision-making, and embedding ethical behavior change principles. Hence, considering interconnected challenges through interdisciplinary collaboration and user-centered design will be important to deploying safe, effective, and just AI tools supporting healthy aging nutrition in the home and clinical settings. To effectively advance artificial intelligence (AI) technologies that promote healthy eating among older adults, it is crucial for both the scientific and international communities to implement a coordinated, ethical, and interdisciplinary approach. This means establishing global research consortia that bring together experts in various fields such as AI technology, nutrition, geriatrics, and behavioral science. By integrating these diverse perspectives, we can develop AI tools that are not only clinically relevant but also culturally responsive to the unique needs of older populations.

One immediate priority is to address privacy concerns associated with data usage. This can be achieved through the creation of transparent data policies that prioritize informed consent processes specifically tailored for older adults. Additionally, employing privacy-preserving techniques, such as anonymization of data, will help protect sensitive information. To minimize biases in AI model training, it is essential to use diverse datasets that reflect a wide range of health profiles, dietary habits, and cultural backgrounds. Furthermore, conducting longitudinal studies and clinical trials will be vital in assessing the real-world impact of these AI interventions, ensuring that they effectively meet the needs of older adults.

Funding agencies have an essential role in this area. They should prioritize projects that seek to harmonize AI technologies with aged care and nutrition strategies. Encouraging open science practices, such as data sharing and transparent reporting, can build trust among stakeholders and enhance the reproducibility of research findings.

The translation of research into practical applications calls for the development of user-friendly AI tools that are co-designed with older adults and their caregivers. Engaging stakeholders, including aged care service providers, nurses, nutritionists, dietitians, and aged care support workers, in the design process will ensure that these tools address real-world challenges. These AI solutions should be seamlessly integrated into clinical workflows and supported by comprehensive training programs that equip healthcare professionals with the necessary skills to utilize them effectively.

Moving beyond technological innovations, AI offers a plethora of additional benefits aimed at enhancing the nutritional well-being of older adults. By promoting independence, AI applications empower seniors to manage their dietary choices proactively, reducing their reliance on caregivers for everyday decisions. Moreover, by providing tools that facilitate social and emotional engagement, AI helps mitigate the feelings of loneliness that can accompany mealtime, fostering a sense of community and connection even in solitary dining situations.

AI’s ability to deliver personalized dietary guidance remotely dramatically expands access to nutritional support, especially in underserved areas where resources may be limited. Such tools can also play a critical role in reinforcing healthy eating habits through timely reminders and motivational feedback, which can encourage long-term behavior change. In addition, by aggregating and analyzing data collected from users, AI systems can provide valuable insights that inform public health strategies and shape policy decisions aimed at improving nutritional outcomes for older adults.

## 6. Limitations of the Review

In this review, we covered different aspects of AI-driven nutritional interventions tailored to support older adults. Although we achieved our overarching aim centered on our key research questions (RQ1–RQ5) to uncover insights that can guide the development of effective and ethically viable interventions, there are a few noteworthy limitations we provide to guide future work. First, our search keywords did not cover individual types of AI interventions such as robots, sensors, machine learning, and other ones we found in our review; this could have limited the number of papers we collected on specific cases. However, we used the AI term to be able to broadly cover these related intervention types. Although this review intended to cover AI broadly, the papers we retrieved focused on a few AI technologies such as sensors, chatbots, ML algorithms, recommender systems, robots, computer vision, expert systems, eye tracking, and speech assistants. While AI is still emerging, we recommend that future work should cover other areas such as large language models, multimodal generative systems, and other related ones.

Third, we focused on a few databases: ACM digital library, IEEE, ScienceDirect, PsycINFO, CINAHL, PubMed, and Scopus. While there are other sources, including search engines like Google Scholar, we excluded Google Scholar because it often produces a large volume of duplicates, including grey literature and non-peer-reviewed sources, and lacks transparent indexing and filtering options, which makes it difficult to ensure the systematic, reproducible searches required in a systematic review. However, we acknowledge that this exclusion may have limited our access to additional papers not indexed in our selected databases. Additionally, we concluded the literature search on 29 April 2025, which may not account for articles published from May to December 2025.

Finally, we focused our search on English papers only. This could have limited the number of papers we collected, suggesting the inclusion of more geographical coverage. However, we will consider the translation of these papers in the future to understand nuances they provide.

## 7. Conclusions

We conducted a review of 30 studies published within the last 10 years (2015–2025) to understand the extent to which AI interventions have been tailored to support the nutritional health and dietary needs of older adults. Drawing on these bodies of knowledge, we synthesized findings across four key dimensions: emerging research trends, intervention effectiveness, methodological and contextual limitations, and ethical considerations underlying the design and deployment of AI-driven solutions for promoting healthy eating in aging populations. Results from our synthesis show that, while the field is still emerging, there has been an increase in research activity since 2020, accompanied by encouraging results in technical performance, user satisfaction, and measurable health outcomes. AI modalities ranging from machine learning algorithms and computer vision systems to chatbots and robotics have shown the capacity to accurately monitor dietary behaviors, personalize recommendations, and support nutrition-related decision making in both experimental and applied settings. Most importantly, our synthesis emphasizes the persistent gaps and challenges that must be addressed before these technologies can achieve widespread, equitable, and sustainable real-world impact. Hence, we conclude that the future of AI-supported nutrition for healthy aging will require research dimensions such as rigorous clinical validation, inclusion of diverse and high-risk nutritional patterns, e.g., choking, integration of multimodal sensing for comprehensive nutritional and safety monitoring and embedding of theory-driven behavioral strategies to sustain change. By grounding the future direction of AI interventions in these dimensions, older adults will meaningfully benefit from enhanced dietary health, safety, and quality of life.

## Figures and Tables

**Table 2:** Summary of the different eating patterns of older adults tracked using AI technologies.

S/N	Reference(s)	Eating Patterns	Underlying Health Condition	AI Technology Used	ML Algorithm	Accuracy	Type of Sensor	Theories and Models	Effective?
1	[70]	Breakfast, lunch, dinner selections and personalized eating preferences considered	Not specified	ML Algorithm	Collaborative Filtering (Item-based, User-based) fused with a custom weighting mechanism	63.1% for fusion model (better than individual methods	X	X	Yes
2	[69]	Mouthful intervals, bite-to-bite analysis	Parkinson’s disease	ML Algorithm	Deep Learning with OpenPose feature extraction + LSTM	Correlation coefficient 0.95 (near-perfect agreement)	X	X	Yes
3	[53]	Family meal processes including breakfast, lunch, and dinner coordination	X	Robots	X	X	X	Feminist theory (Marjorie DeVault—Feeding as Care Work), Sensitizing Concepts (Generative and Systemic Discontents)	Potentially effective
4	[51]	Meal intake patterns, Health-related meal behaviors	Lonely death risk, Social isolation, Chronic health issues	Chatbots	X	X	X	Self-Disclosure Theory	Yes
5	[63]	Meal-based food logging (breakfast, lunch, dinner, snacks)	Prediabetes, Type 2 Diabetes prevention	Sensors	X	X	GSM-based body scale, Fitness band (similar to Fitbit)	X	Yes
6	[71]	Three-course meals (appetizer, main dish, dessert)	Public health promotion, healthy eating, chronic disease prevention (e.g., obesity, diabetes)	Recommender systems	X	Best Recall@20 = 0.3753 (CrossCBR model on MealRec+H)	X	Cooperative Interaction Learning, Collaborative Filtering, Multi-task Learning, Mutual Learning, Contrastive Learning	Yes
7	[72]	Meal preparation tracked (as high-level daily activity)	Aging, Mild cognitive impairment	Sensors	X	Semi-supervised WF1 over 90% for older adults with 50% labeled data	Passive Infrared (PIR) motion sensors, Contact sensors	Self-Supervised Learning (SSL), SimCLR Framework, Attention Mechanism	Yes
8	[73]	Monitored through personalized nutrition planning	Aging, Chronic disease prevention (e.g., diabetes, cardiovascular disease)	Sensors	X	X	Wearable sensors (e.g., smartwatches, fitness trackers), Genetic sequencing sensors	Smart Nutrition Model	Potentially effective (needs empirical validation)
9	[74]	Adapted based on seasonal changes and health needs	Aging, Risk of dehydration, Hypothermia, Emotional well-being	Robot/ML algorithm	LSTM (speech recognition), CNN (emotion recognition)	Emotion recognition model average accuracy: 84.0%	X	X	Yes
10	[75]	Daily meal decisions and healthy food recognition supported	Type 2 Diabetes	Chatbot	X	X	X	Retrieval-Based Chatbot Model	Yes
11	[76]	Monitored through dietary intake tracking and recommendations	Chronic diseases, Social isolation, Physical health decline	Sensors	X	X	Health monitoring sensors, Environmental sensors	X	Yes
12	[77]	Healthy eating promotion integrated into dialogue	Social isolation, Depression, Loneliness	Chatbots	X	X	X	Large Language Models (LLMs), Personalized Memory Mechanisms	Potentially effective
13	[66]	Eating behavior analyzed by gesture intervals (hand-to-mouth actions)	Obesity, Malnutrition in older adults	Sensors	X	Segmental F1-score of 0.944 at IoU 0.5 on Huashan dataset	Wrist-mounted and Head-mounted IMU sensors	Multistage Temporal Convolutional Network (MS-TCN), Sequence-to-Sequence (Seq2Seq) modeling	Yes
14	[67]	Based on Mediterranean diet food categories (breakfast, lunch, dinner patterns)	Type 1 Diabetes	Computer Vision	X	82.4% Top-1 accuracy, 97.5% Top-5 accuracy, 10.7% MAPE for volume estimation	X	X	Yes
15	[78]	Meal completeness, intake variability, dietary behavior patterns analyzed	Frailty, Cognitive impairments, Malnutrition	ML Algorithm	Logistic Regression with LASSO, Random Forest, AB, RUSBoost, Support Vector Machines, k-Neural Network, CART	94% accuracy, 92% recall with combined nutrition and body composition data	X	Deep Learning (EfficientNet-B2), Stereo-vision for Volume Estimation	Yes
16	[60]	Breakfast, lunch, dinner recommendations based on personal health profile	Aging, Chronic diseases (e.g., diabetes, heart disease, hypertension, stroke)	Chatbot	X	Precision = 87.5%, Recall = 100%, F1-Score = 93.3%	X	Ontology, Semantic Web Rule Language (SWRL)	Yes
17	[68]	Detected using unsupervised clustering, based on motion and contact sensor data	Frailty, Poor nutrition in elderly	Sensors/Clustering Algorithm	K-Means, GMM, DBSCAN clustering	K-Means showed best performance for identifying meal-taking activities based on lowest Davies-Bouldin score of 0.604	Passive Infrared (PIR) motion sensors, Contact sensors	X	Yes
18	[62]	Not Explicit	Age-related disability, Neurological disorders, Hand amputees	Robot/ML Algorithms	Genetic algorithms	0.67% overshoot (minimal positional error)	X	Fractional Order PID (FOPID), Genetic Algorithm (GA) Optimization, Forward Kinematics (PoE Method), Inverse Kinematics (Newton-Raphson Method), Fuzzy PID Control	Yes
19	[61]	Not explicitly detailed as eating patterns but focused on enabling self-feeding tasks	Malnutrition prevention, Aging-related nutritional challenges	Chatbot	X	X	X	Participatory Design	Moderately effective (improved in second version with proactive features)
20	[57].	Not explicitly discussed (focus on nutrient intake)	Accelerated Vascular Aging (EVA)	Sensors	X	68.80%	SphygmoCor^®^ for cfPWV (AtCor Medical Pty Ltd., Head Office, West Ryde, Austalia), Sonosite Micromax^®^, (Sonosite, Inc., Bothell, WA, USA)for cIMT	X	Moderately effective
21	[59]	Three meals per day recommendations aligned with traditional Chinese meal structure	Multi-chronic conditions (hypertension, diabetes, dyslipidemia)	Recommender systems	X	X	X	Knowledge Graph (KG) and Rule-based Filtering	Yes
22	[58]	Three meals per day recommendations aligned with traditional Chinese meal structure	Dysphagia (swallowing difficulties, common in elderly and neurological diseases)	Sensors	Support Vector Machine (SVM) with Gaussian Kernel	95.07%	Refitted Laryngeal Bone Conduction Headset (PTE-796) as vibration sensor	X	Yes
23	[56]	Daily meal logging, food categories based on dietary habits	Geriatric frailty syndrome, risk of malnutrition	Computer Vision	SSD-MobileNet V2	Object detection rate: 61% (direct), 91% (with interview mode)	X	X	Yes
24	[64]	Daily meal plan creation with food categories	Elderly nutritional deficiencies, Diabetes, Hypertension (user-dependent personalized constraints)	Recommender systems	Particle Swarm Optimization (Metaheuristic)	Final fitness values ≈ 0.9999 after ~30 iterations in experiments	X	Multi-Constraint Particle Swarm Optimization (PSO), Rule-based filtering	
25	[79]	Not discussed explicitly; focus on food product nutrition information	Age-related sensory decline (vision, hearing), Technological unfamiliarity	Computer Vision, Speech assistant	Kaldi for ASR, Rasa NLU for intent recognition	Qualitative results only; acceptable recognition in quiet environments	X	Privacy-by-Design Framework, Open Source Architecture (Kaldi ASR, Rasa NLU, MaryTTS)	Yes
26	[54]	Breakfast meal selection based on diabetes-appropriate food recommendation	Type II Diabetes management in newly diagnosed older adults	Eye tracking	X	X	Eye tracker (for a subset of participants); otherwise, user interaction logs	Technology Acceptance Model (TAM), Ageing-Centered Design Principles	Yes
27	[65]	Full-day meal pattern (breakfast, morning snack, lunch, afternoon snack, dinner)	Age-related chronic diseases prevention and healthy aging	ML Algorithm	Hybrid Metaheuristics (no direct ML models; combines evolutionary computing and memory-based learning)	Fitness values mostly >0.90; execution times reduced by ~80–90% compared to baseline	X	Hybrid Clonal Selection Algorithm (CLONALG + Flower Pollination + Tabu Search + Reinforcement Learning)	Yes
28	[80]	Daily meal patterns analyzed (energy, macro and micronutrient intake)	Malnutrition risk, Diabetes, Cardiovascular diseases, Obesity	Expert system	X	X	X	X	Yes
29	[81]	Daily and weekly eating pattern monitoring for nutritional adequacy	Malnutrition risk, Nutrient deficiency, Dietary restrictions (e.g., allergies, religious restrictions)	Recommender systems	X	X	X	X	Yes
30	[82]	Focused on nocturnal glucose fluctuations rather than eating patterns	Hypoglycemia (low blood glucose)	Sensors	X	~90% sensitivity and specificity for hypoglycemia detection	Wearable ECG sensor (Zephyr BioPatch), Continuous Glucose Monitor (CGM)	X	Yes

## Data Availability

No new data were created or analyzed in this study.

## Appendix A

**Table A1:** Summary of tracked eating patterns, associated health conditions, and effectiveness of AI-based interventions.

S/N	Author(s)	Year	Summary of Eating Patterns Tracked	Underlying Conditions	Types of AI Technologies	Effective?
1	Wang et al. [70]	2023	Yes, focus on meal recommendations (breakfast, lunch, dinner) for seniors	Not specified	ML Algorithm	Yes
2	Ioakeimidis et al. [69]	2024	Meal analysis focusing on mouthfuls and bite detection	Parkinson’s disease	ML Algorithm	Yes
3	Wu et al. [53]	2024	Yes, exploration of family meals including planning, purchasing, cooking, eating, and cleaning	Not specified	Robot	Conceptual effectiveness discussed; no technology deployed yet
4	Jo et al. [51]	2024	Yes, users disclosed meal habits (e.g., meal skipping, meal quality)	Lonely death risk, Social isolation, Chronic health issues	Chatbot	Yes, LTM led to greater self-disclosure and positive emotional responses
5	Nolting et al. [63]	2019	Food logging integrated into the program, focusing on meal intakes	Prediabetes, Type 2 Diabetes prevention	Sensors	Yes (41% achieved 5%+ weight loss in treatment)
6	Li et al. [71]	2024	Extensive discussion about user-course and user-meal interactions for meals including breakfast, lunch, and dinner	Public health promotion, healthy eating, chronic disease prevention (e.g., obesity, diabetes)	Recommender systems	Yes
7	Chen et al. [72]	2024	Activities like preparing meals are labeled in the LOADA app	Aging, Mild cognitive impairment	Sensors	Yes
8	Armand et al. [32]	2024	Yes, focus on personalized nutrition intake tracking through wearables and smart recommendations	Aging, Chronic disease prevention (e.g., diabetes, cardiovascular disease)	Sensors	Potentially effective (needs empirical validation)
9	Yao et al. [74]	2024	Yes, through climate and seasonal recommendations for clothing and dietary intake	Aging, Risk of dehydration, Hypothermia, Emotional well-being	Robot	Yes
10	Cheng et al. [75]	2018	Yes, includes assessing healthy eating behaviors and food-related queries	Type 2 Diabetes management	Chatbot	Yes
11	Kiran et al. [76]	2024	Yes, dietary tracking and personalized nutrition plans are part of the system	Chronic diseases, Social isolation, Physical health decline	Sensors	Yes
12	So et al. [77]	2025	Yes, promoting healthy living through conversations about nutrition and physical activity	Social isolation, Depression, Loneliness	Chatbot	Potentially effective (but limited adoption in initial pilot)
13	Wang et al. [66]	2024	Yes, continuous detection of eating gestures implies food intake	Obesity, Malnutrition in older adults	Sensors	Yes
14	Konstantakopoulos et al. [67]	2022	Yes, food intake analyzed via image-based dietary assessment	Type 1 Diabetes management	Computer Vision	Yes
15	Martino et al. [78]	2021	Yes, dietary intake monitored and analyzed via DEW application	Frailty, Cognitive impairments, Malnutrition	ML Algorithm	Yes
16	Prasetyo et al. [60]	2024	Yes, based on personalized daily meal plans	Aging, Chronic diseases (e.g., diabetes, heart disease, hypertension, stroke)	Chatbot	Yes
17	Derouiche et al. [68]	2024	Yes, focus on detecting meal-taking activities	Frailty, Poor nutrition in elderly	Sensors	Yes
18	Parikh et al. [62]	2024	Yes, robot assists elderly/specially abled people during feeding activities	Age-related disability, Neurological disorders, Hand amputees	Robot	Yes
19	Weber et al. [61]	2023	Yes, users tracked food intake, requested recipes, and logged weight	Malnutrition prevention, Aging-related nutritional challenges	Chatbot	Moderately effective (improved in second version with proactive features)
20	Patino-Alonso et al. [57]	2023	Yes, food intake analyzed via macronutrient and micronutrient consumption data	Accelerated Vascular Aging (EVA)	Sensors	Moderately effective
21	Xu et al. [59]	2024	Yes, through dish recommendation and personalized combo meals	Multi-chronic conditions (hypertension, diabetes, dyslipidemia)	Recommender systems	Yes
22	He et al. [58]	2022	Indirectly implied via swallowing function and dysphagia screening	Dysphagia (swallowing difficulties, common in elderly and neurological diseases)	Sensors	Yes
23	Elfert et al. [56]	2021	Yes, nutrition diary entries for all meals and supplements	Geriatric frailty syndrome, risk of malnutrition	Computer Vision	Yes
24	Gautam and Gulhane [64]	2021	Yes, through generation of daily meal plans including breakfast, lunch, snacks, dinner	Elderly nutritional deficiencies, Diabetes, Hypertension (user-dependent personalized constraints)	Recommender systems	Yes (in initial experiments)
25	Seiderer et al. [79]	2020	Yes, nutrition information retrieval for food products	Age-related sensory decline (vision, hearing), Technological unfamiliarity	Computer Vision, Speech assistant	Yes
26	Chao and Hass [54]	2020	Yes, users select breakfast cereals considering health recommendations	Type II Diabetes management in newly diagnosed older adults	Eye tracking	Yes (for Nutri-score labeling format)
27	Chifu et al. [65]	2016	Yes, daily menu plans were generated including food items for breakfast, lunch, dinner, and snacks	Age-related chronic diseases prevention and healthy aging	ML Algorithm	Yes
28	Cioara et al. [80]	2018	Yes, daily intake monitoring and nutrient evaluation	Malnutrition risk, Diabetes, Cardiovascular diseases, Obesity	Expert system	Yes
29	Espín et al. [81]	2015	Yes, weekly diet plans based on food intake and nutrient tracking	Malnutrition risk, Nutrient deficiency, Dietary restrictions (e.g., allergies, religious restrictions)	Recommender systems	Yes (prototype phase)
30	Porumb et al. [82]	2020	Indirectly implied through glucose monitoring; not specific to meals	Hypoglycemia (low blood glucose)	Sensors	Yes
